# Supplementary material for: Long-term elevated levels of loneliness are linked to lower health-related quality of life in middle-aged Australian women
Source: Commun Psychol. 2025 May 26;3:85. doi: 10.1038/s44271-025-00264-z (PMC12106689; doi:10.1038/s44271-025-00264-z)
Supplement: Supplementary file 2 — Supplementary Information [file 44271_2025_264_MOESM2_ESM.pdf]

Long-term elevated levels of loneliness are linked to lower health-related quality of life in middle-aged Australian women - Supplementary Information

Table of Contents

1. STROBE reporting 3

2. Supplementary Notes 6

3. Additional results 11

4. Sensitivity analyses 18

Tables

Table S1.1. Statement- Checklist of items that should be included in reports of cohort studies 3

The datasets for the additional results are available at: Australian Longitudinal Study on Women's Health. *Applying for the ALSWH full dataset and linked data*, https://alswh.org.au/for-data-users/applying-for-data/full-dataset-and-linked-data/ 11

Table S3.1. Number of participants according to wave and the total number of participants included in the analysis 11

Table S3.2. Criteria for selecting the number of trajectories (waves 3-8) 12

Table S3.3. Multivariate linear regression of the predictors of the physical component summary (PCS) and mental component summary (MCS) (n = 13,714) 13

The datasets for the sensitivity analysis are available at: Australian Longitudinal Study on Women's Health. *Applying for the ALSWH full dataset and linked data*, https://alswh.org.au/for-data-users/applying-for-data/full-dataset-and-linked-data/ 18

Table S4.1. Sensitivity analysis: Multivariate multinomial logistic regression predicting latent class membership using baseline predictors- Probit model results (n = 13,714) 18

Table S4.2. Sensitivity analysis: Multivariate multinomial linear regression predicting latent class membership using baseline predictors (Beta coefficients, 95%CI): Data fully adjusted,

adjusted only for sociodemographic characteristics and unadjusted- Probit model results (n = 13,714) 21

Table S4.3. Sensitivity analysis: criteria for selecting the number of trajectories (waves 3-8) using a new cut-point for loneliness (1. Rarely or none of the time vs. levels 2-4; n = 13,714) 23

Table S4.4. Sensitivity analysis: Multivariate multinomial logistic regression predicting latent class membership using baseline predictors using a new cut-point for loneliness (1. Rarely or none of the time vs. levels 2-4; n = 13,714) 24

Table S4.5. Sensitivity analysis: multivariate multinomial linear regression predicting latent class membership using baseline predictors (Beta coefficients, 95%CI) using a new cut-point for loneliness (1. Rarely or none of the time vs. levels 2-4): Data fully adjusted, adjusted only for sociodemographic characteristics and unadjusted (n = 13,714).......................................27

Figures

Figure S3.1. Q-Q plot for assessing normality of standardised residuals...............................15

Figure S3.2. Summary of missing data in each analysis variable..16

Figure S3.3. Common patterns of missing data...17

1. STROBE reporting

Table S1.1. Statement- Checklist of items that should be included in reports of cohort studies

	Item No	Recommendation	Page No
**Title and abstract**	1	(*a*) Indicate the study's design with a commonly used term in the title or the abstract	2
		(*b*) Provide in the abstract an informative and balanced summary of what was done and what was found	2
**Introduction**			
Background/rationale	2	Explain the scientific background and rationale for the investigation being reported	3-4
Objectives	3	State specific objectives, including any prespecified hypotheses	4
**Methods**			
Study design	4	Present key elements of study design early in the paper	5
Setting	5	Describe the setting, locations, and relevant dates, including periods of recruitment, exposure, follow-up, and data collection	5
Participants	6	(*a*) Give the eligibility criteria, and the sources and methods of selection of participants. Describe methods of follow-up	5
		(*b*) For matched studies, give matching criteria and number of exposed and unexposed	
Variables	7	Clearly define all outcomes, exposures, predictors, potential confounders, and effect modifiers. Give diagnostic criteria, if applicable	5-7
Data sources/ measurement	8*	For each variable of interest, give sources of data and details of methods of assessment (measurement). Describe comparability of assessment methods if there is more than one group	5-7
Bias	9	Describe any efforts to address potential sources of bias	7-9
Study size	10	Explain how the study size was arrived at	9, Table S3.1
Quantitative variables	11	Explain how quantitative variables were handled in the analyses. If applicable, describe which groupings were chosen and why	5-7, Appendix 2,

Statistical methods	12	(*a*) Describe all statistical methods, including those used to control for confounding	7-9
		(*b*) Describe any methods used to examine subgroups and interactions	9
		(*c*) Explain how missing data were addressed	9, Figures S3.2-S3.3
		(*d*) If applicable, explain how loss to follow-up was addressed	9, Figures S3.2-S3.3
		(*e*) Describe any sensitivity analyses	9
**Results**			
Participants	13*	(a) Report numbers of individuals at each stage of study—eg numbers potentially eligible, examined for eligibility, confirmed eligible, included in the study, completing follow-up, and analysed	5, 9, Table S3.1
		(b) Give reasons for non-participation at each stage	9, Table S3.1
		(c) Consider use of a flow diagram	Table S3.1
Descriptive data	14*	(a) Give characteristics of study participants (eg demographic, clinical, social) and information on exposures and potential confounders	9-10, Table 1
		(b) Indicate number of participants with missing data for each variable of interest	Figures S3.2-S3.3
		(c) Summarise follow-up time (eg, average and total amount)	Table S3.1
Outcome data	15*	Report numbers of outcome events or summary measures over time	10-12
Main results	16	(*a*) Give unadjusted estimates and, if applicable, confounder-adjusted estimates and their precision (eg, 95% confidence interval). Make clear which confounders were adjusted for and why they were included	10-12, Tables 2-3, Table S3.3
		(*b*) Report category boundaries when continuous variables were categorized	10-12, Tables 2-3, Table S3.3
		(*c*) If relevant, consider translating estimates of relative risk into absolute risk for a meaningful time period	-
Other analyses	17	Report other analyses done—eg analyses of subgroups and interactions, and sensitivity analyses	13, Table 3, Tables S4.1-S4.5
**Discussion**			
Key results	18	Summarise key results with reference to study objectives	13

Limitations	19	Discuss limitations of the study, taking into account sources of potential bias or imprecision. Discuss both direction and magnitude of any potential bias	15-16
Interpretation	20	Give a cautious overall interpretation of results considering objectives, limitations, multiplicity of analyses, results from similar studies, and other relevant evidence	13-15
Generalisability	21	Discuss the generalisability (external validity) of the study results	15-16
**Other information**			
Funding	22	Give the source of funding and the role of the funders for the present study and, if applicable, for the original study on which the present article is based	17

*Give information separately for exposed and unexposed groups.

Note: An Explanation and Elaboration article discusses each checklist item and gives methodological background and published examples of transparent reporting. The STROBE checklist is best used in conjunction with this article (freely available on the Web sites of PLoS Medicine at http://www.plosmedicine.org/, Annals of Internal Medicine at http://www.annals.org/, and Epidemiology at http://www.epidem.com/). Information on the STROBE Initiative is available at http://www.strobe-statement.org.

2. Supplementary Notes

Baseline predictors

Marital status

Marital status was measured by asking participants about their current marital status. Answers included: 1. married, de Facto/opposite sex, de Facto/same sex, 2. separated, divorced, widowed, and 3. single. We recoded the answers into the following categories: 1. partnered, 2. not partnered, 3. widowed.

Index of Relative Socio-Economic Disadvantage (IRSD)

The residential areas of participants were analysed by the Index of Relative Socio-Economic Disadvantage (IRSD) from the Socio-Economic Indexes for Areas (SEIFA) (1). This is a population-based metric, evaluating the relative disadvantage of geographic regions by considering multiple socioeconomic factors including household income, educational attainment, occupational status, housing costs (rent and mortgage payments), dwelling size, occupancy, and disability rates. The data used for this index is obtained from the Australian Census, providing a comprehensive snapshot of the socio-economic conditions prevalent in each area (2).

Area remoteness

The Accessibility-Remoteness Index of Australia Plus (ARIA+) (3) was used to examine the geographical remoteness of participants. This index is a classification system based on the Australian Statistical Geography Standard (ASGS) from the Australian Census (4). Utilizing participants' residential postcodes, they were categorized into one of three groups: major city, regional, or remote.

Employment status

In all waves except the second one, participants were asked about their primary current occupation. The response options included roles such as manager, professional, paraprofessional, trade, admin. assist, sales/service, machine operator, manual worker, never engaged in paid work, and other. The responses to this question were classified, with a designation of 0 indicating "not employed" and 1 denoting "employed."

In the second wave, participants were presented with two distinct inquiries regarding their primary and secondary occupational statuses. The questions prompted them to specify the nature of their main and secondary occupations, with response options such as no secondary

occupation, full-time, part-time/casual, work without pay, home duties, studying, unemployed-looking, unpaid voluntary, retired, and unable to work. All unpaid occupations, including home duties, studying, retirement, etc., were collectively treated as unpaid work and recategorized as 0, indicating unemployed. both full-time and part-time positions were classified as paid work and recoded as 1, representing employed status.

Living alone

Participants indicated whether they lived alone with response options coded as 0 = no, 1 = yes. However, in the second wave, participants were asked to specify the number of people living with them, including categories such as partner/spouse, children under 16 years, children 16-18 years, children over 18 years, parents, other adult relatives, and other non-family members. The responses to this question were subsequently recoded, with a value of 0 denoting "not live alone" and 1 signifying "live alone."

Country of birth

Participants were inquired about their country of birth, with response options including Australian born, other English-Speaking Background, Europe, Asia, and Other. These responses were subsequently categorized into a binary variable, distinguishing between individuals who were Australian born and those who were not Australian born.

Language spoken at home

Participants were surveyed about the language spoken at home. Potential responses included English, Aust, English, Other, European, Asian, and Other. Subsequently, a variable was derived from this information, categorizing participants into four distinct levels based on the language spoken at home: English, European, Asian, and Other.

Level of education

Education levels were assessed based on the highest qualification completed by participants, with potential responses ranging from 'no formal qualification' to 'higher university degree'. For the purpose of analysis, these responses were recategorized into a three-level variable: 1 = high school or less, 2 = tertiary not university, 3 = college/university.

Alcohol consumption

The assessment of alcohol consumption adhered to the revised Australian guidelines introduced in 2020. These guidelines suggest that adults should limit their alcohol intake to no more than 10 standard drinks per week and no more than four standard drinks on any

single day (5). Participants were asked to specify the frequency of their alcohol consumption, ranging from 'never' to 'every day.' Additionally, participants indicated the typical number of drinks they usually have in a day, with response options ranging from one to more than nine drinks, and the frequency with which they engage in heavy episodic drinking, defined as consuming five or more drinks on a single occasion, using a scale from 'never drink' to 'more than once a week.' Participants reported the consumption of more than 10 standard drinks in a week or more than four standard drinks on any one day or if they consumed more than five drinks at a time, they were defined as being at ‘levels that increase the risk of alcohol-related disease or injury’.

Smoking

Participants' smoking status was assessed using the Australian Institute of Health and Welfare measure, which includes response options such as never smoker, ex-smoker, irregular smoker, weekly smoker, and daily smoker (6). The responses were subsequently recategorized into three broader categories: 0 for "never smoked," 1 for "ex-smoker," and 2 for "currently smoking."

Body Mass Index

Participants provided self-reported data on their height and weight, from which their Body Mass Index (BMI) was calculated. The BMI values were then classified into four levels according to the World Health Organization guidelines:

1) Underweight (BMI < 18.5)
2) Healthy weight (18.5 <= BMI < 25)
3) Overweight (25 <= BMI < 30)
4) Obese (30 <= BMI)

Anxiety and depression diagnoses

Participants were asked about whether they had received a diagnosis or treatment for various physical and mental conditions within the past three years. For the purposes of the current study, the focus was specifically on the items related to diagnoses of anxiety and depression. The response options for each of the two variables were 0. never, 1. last two years, 2. >two years ago, 3. Both. The responses were recoded as 0 for "never" and 1 for "ever". Participants were also asked about having symptoms of anxiety and depression on a scale of never-often. Responses for each of the two variables were recoded as 0 = never/rarely, 2 = sometimes/often. New variables were constructed for anxiety and depression with those ever

having a diagnosis or experiencing symptoms sometimes/often categorised as having the condition.

Depression

The Centre for Epidemiologic Studies Depression (CES-D) Scale was used to examine depression. Participants were asked about the ways they felt or behave during the past week using 10 items on a 4-point Likert scale (0 = Rarely or none of the time, 3 = Most or all of the time) (7).

Stress

Stress levels were assessed using the Perceived Stress Questionnaire (8). This measure is derived from the average of 10 items, each rated on a scale ranging from 0 (not at all stressed) to 4 (extremely stressed). The items encompass various sources of stress, including but not limited to health, work, money, and relationships. The cumulative score provides an overall indication of participants' perceived stress levels across multiple dimensions.

Social support

From Survey 2 onwards, social support was obtained using the 6-item Medical Outcomes Study (MOS) scale, which is an abbreviated version of the larger 19-item MOS support index (9, 10). This scale assesses various domains of social support, including tangible support, affectionate support, positive social interaction, and emotional/informational support. Participants provided responses on a scale ranging from 1 (none of the time) to 5 (all of the time), where a higher score indicates a higher level of perceived support.

Supplementary references

1. Australian Bureau of Statistics. Technical Paper: Socio-Economic Indexes for Areas (SEIFA). 2016.
2. Pink B. Information paper: An introduction to Socio-Economic Indexes for Areas (SEIFA) 2006. Information paper: An introduction to Socio-Economic Indexes for Areas (SEIFA) 2006. Canberra: Australian Bureau of Statistics, Commonwealth of Australia; 2008. Contract No.: ABS Catalogue No. 2039.0.
3. Glover JD, Tennant SK. Remote areas statistical geography in Australia: notes on the Accessibility/Remoteness Index for Australia (ARIA+ version): Public Health Information Development Unit, The University of Adelaide; 2003.

4. Australian Bureau of Statistics. The Australian statistical geography standard (ASGS) remoteness structure. Australian Bureau of Statistics Canberra; 2018.
5. Australian Government National Health and Medical Research Council. Australian guidelines to reduce health risks from drinking alcohol. 2020 [Available from: https://www.nhmrc.gov.au/file/15923/download?token=t0Hrxdvq.
6. AIHW. (2021). Australian Burden of Disease Study 2018: Key findings. Canberra: Australian Institute of Health and Welfare. Retrieved from https://www.aihw.gov.au/reports/burden-of-disease/burden-of-disease-study2018-key-findings/contents/about
7. Radloff LS. The CES-D Scale: A self-report depression scale for research in the general population. Applied Psychological Assessment. 1977;1:385-401.
8. Bell S, Lee C. Development of the Perceived Stress Questionnaire for Young Women. Psychology, Health Med. 2002;7(2):189-201.
9. Ware JE, Sherbourne CD. The MOS 36-item short-form health survey (SF-36). Conceptual framework and item selection. Med Care. 1992;30(6):473–483.
10. Holden, L., Lee, C., Hockey, R., Ware, R. S., & Dobson, A. J. (2014). Validation of the MOS Social Support Survey 6-item (MOS-SSS-6) measure with two large population-based samples of Australian women. Qual Life Res, 23(10), 2849- 2853. doi:10.1007/s11136-014-0741-5.

3. Additional results

The datasets for the additional results are available at: Australian Longitudinal Study on Women's Health. *Applying for the ALSWH full dataset and linked data*, https://alswh.org.au/for-data-users/applying-for-data/full-dataset-and-linked-data/

Table S3.1. Number of participants according to wave and the total number of participants included in the analysis

Wave	n
Wave 1	13,714
Wave 2	12,338
Wave 3	11,226
Wave 4	10,905
Wave 5	10,638
Wave 6	10,011
Wave 7	9151
Wave 8	8622
Wave 9	7956
Sample size included in the analysis	13,714

Table S3.2. Criteria for selecting the number of trajectories (waves 3-8)

**Model**	**AIC**	**BIC**	**SSABIC**	**Model group membership proportion**				
				**(1)**	**(2)**	**(3)**	**(4)**	**(5)**
**Overall loneliness**								
**1 class**	38454.3	38498.4	38479.3	100.0%				
**2 class**	34955.5	35051.1	35009.8	84.9%	15.1%			
**3 class**	34836.4	34983.6	34920.0	71.6%	4.4%	24.0%		
**4 class**	**34796.1**	**34994.7**	**34908.9**	**73.8%**	**5.3%**	**17.1%**	**3.7%**	
**5 class**	34785.2	35035.3	34927.3	68.2%	3.7%	1.7%	20.9%	5.5%

Abbreviations: AIC = Akaike information criterion; BIC = Bayesian information criterion; SSABIC = sample size adjusted BIC.

Table S3.3. Multivariate linear regression of the predictors of the physical component summary (PCS) and mental component summary (MCS) (n = 13,714)

	**PCS**<br>**B (95%CI)**	**MCS**<br>**B (95%CI)**
**Loneliness classes**		
Stable-low	Reference	Reference
Increasing	**-1.06 (-2.11, -0.02)**	**-3.73 (-5.42, -2.04)**
Stable-medium	-0.83 (-2.01, 0.35)	**-3.12 (-5.08, -1.15)**
Stable-high	-0.78 (-1.87, 0.31)	**-5.67 (-6.84, -4.49)**
**Risky alcohol consumption** [1]		
No	Reference	Reference
Yes	0.22 (-0.37, 0.80)	-0.09 (-0.60, 0.41)
**Binge drinking**		
No	Reference	Reference
Yes	0.20 (-0.27, 0.67)	0.19 (-0.20, 0.57)
**Smoking**		
Non-smoker	Reference	Reference
Ex smoker	0.06 (-0.43, 0.55)	-0.31 (-0.73, 0.11)
Current smoker	**-1.43 (-2.09, -0.78)**	-0.35 (-0.90, 0.19)
**Baseline PCS**	**0.37 (0.34, 0.40)**	**0.11 (0.08, 0.13)**
**Baseline MCS**	**0.10 (0.07, 0.13)**	**0.19 (0.16, 0.22)**
**BMI**		
Normal weight	Reference	Reference
Underweight	-0.14 (-2.11, 1.82)	0.53 (-1.30, 2.35)
Overweight	**-2.15 (-2.61, -1.70)**	-0.20 (-0.67, 0.27)
Obese	**-4.90 (-5.51, -4.29)**	**-0.49 (-0.97, -0.003)**
**CES-D (Depression)**	-0.05 (-0.11, 0.02)	**-0.11 (-0.17, -0.05)**
**Stress**	**-0.76 (-1.31, -0.20)**	**-1.04 (-1.57, -0.51)**
**Ever had depression** [2]		
No	Reference	Reference
Yes	-0.29 (-0.87, 0.29)	**-1.24 (-1.78, -0.70)**
**Ever had anxiety** [3]		
No	Reference	Reference
Yes	-0.04 (-0.61, 0.53)	-0.51 (-1.03, 0.02)
**Language**		
English	Reference	Reference
European	-0.61 (-1.75, 0.53)	-0.66 (-1.71, 0.38)
Asian	-0.99 (-2.72, 0.74)	0.18 (-1.63, 1.99)
other	-0.26 (-2.40, 1.89)	0.78 (-1.16, 2.73)
**Age**	**-0.19 (-0.32, -0.06)**	0.004 (-0.11, 0.12)
**Area-level socioeconomic status**	**0.004 (0.001, 0.01)**	0.001 (-0.002, 0.005)
**Marital status**		

	PCS<br>B (95%CI)	MCS<br>B (95%CI)
Married	Reference	Reference
Not married	-0.18 (-0.84, 0.48)	0.32 (-0.31, 0.95)
Widower	-0.18 (-1.52, 1.16)	0.45 (-0.88, 1.79)
**Area remoteness**		
Major city	Reference	Reference
Regional	-0.17 (-0.63, 0.29)	-0.01 (-0.42, 0.41)
Remote	-0.57 (-1.58, 0.45)	-0.01 (-0.83, 0.80)
**Employment**		
Not employed	Reference	Reference
Employed	0.40 (-0.10, 0.89)	**0.49 (0.005, 0.97)**
**Country of birth**		
Australia	Reference	Reference
Other	0.22 (-0.24, 0.68)	-0.22 (-0.68, 0.25)
**Education**		
High school and below	Reference	Reference
Trade/apprentice/certificate/diploma	-0.001 (-0-.47, 0.47)	0.37 (-0.07, 0.81)
University	**0.59 (0.06, 1.12)**	0.19 (-0.32, 0.70)
**Living arrangement**		
Live alone	Reference	Reference
Live with someone	0.81 (-0.40, 2.02)	-0.50 (-1.56, 0.56)
**MOS social support**	0.21 (-0.02, 0.44)	**0.42 (0.21, 0.63)**

Note.

Bold text indicates significant findings.

[1] Risky alcohol consumption: consuming more than 10 standard drinks in a week or more than four standard drinks per day.

[2] Depression: ever being told by a doctor about having depression or having symptoms of depression.

[3] Anxiety: ever being told by a doctor about having anxiety or having symptoms of anxiety.

Figure S3.1. Q-Q plot for assessing normality of standardised residuals

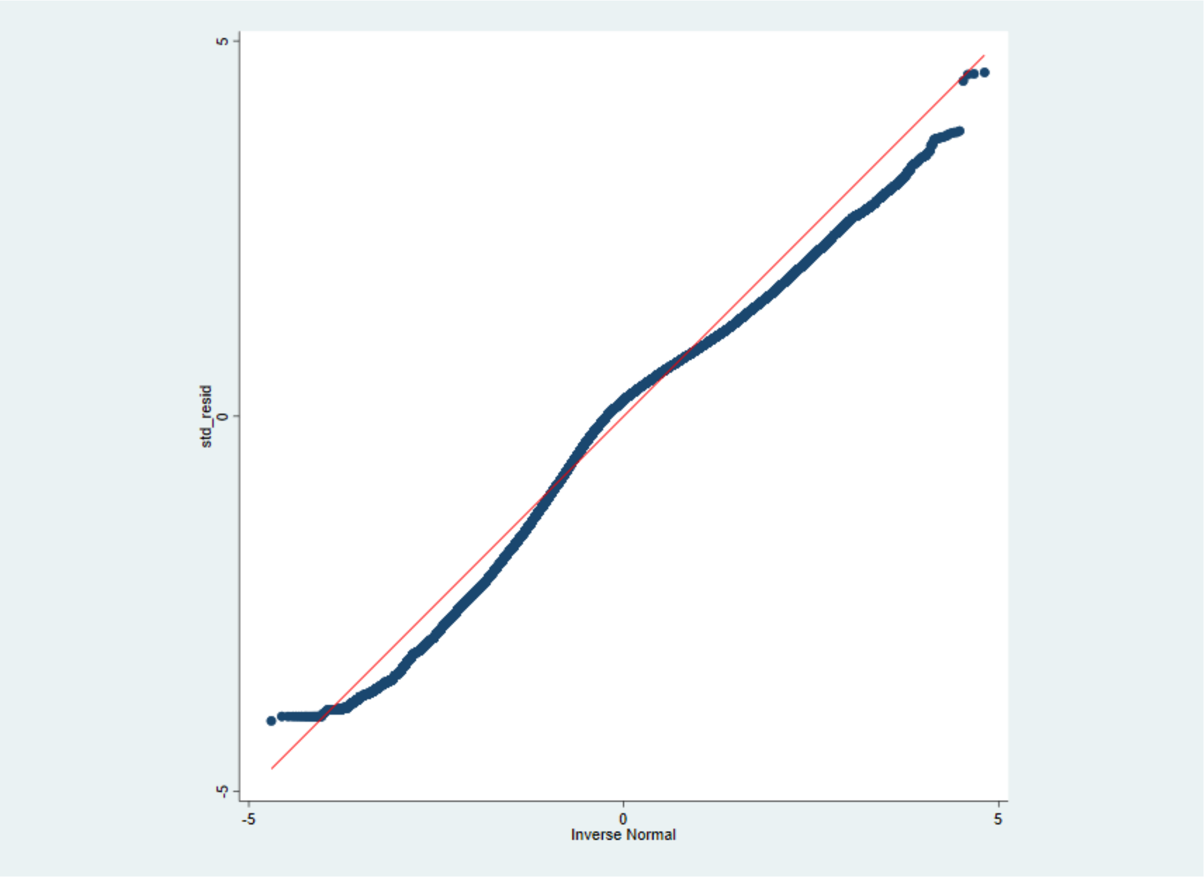

Figure S3.2. Summary of missing data in each analysis variable

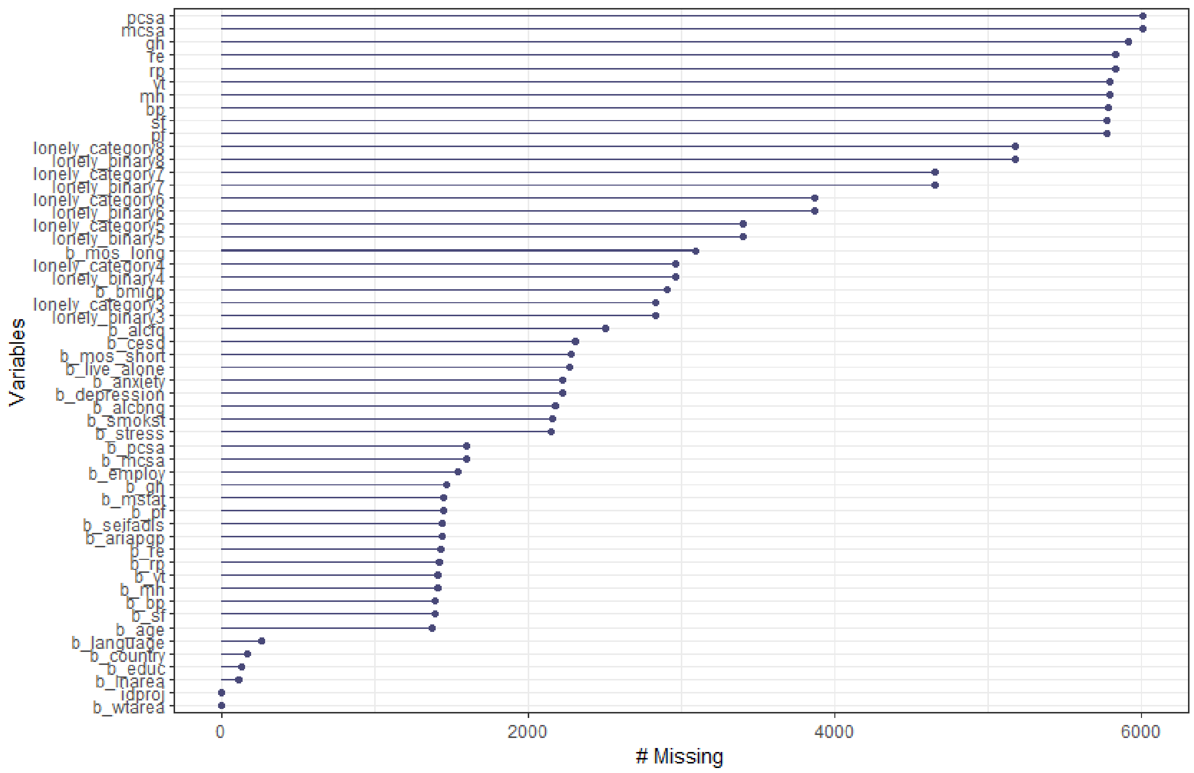

Figure S3.3. Common patterns of missing data

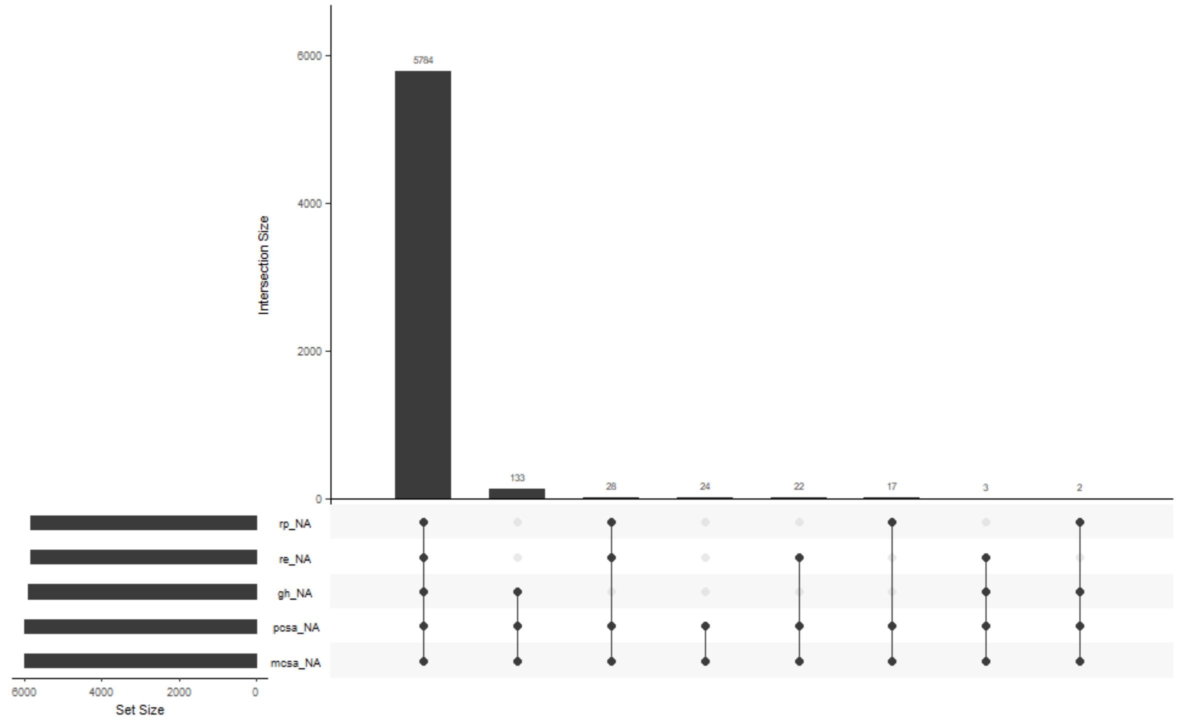

4. Sensitivity analyses

The datasets for the sensitivity analysis are available at: Australian Longitudinal Study on Women's Health. *Applying for the ALSWH full dataset and linked data*, https://alswh.org.au/for-data-users/applying-for-data/full-dataset-and-linked-data/

Table S4.1. Sensitivity analysis: Multivariate multinomial logistic regression predicting latent class membership using baseline predictors- Probit model results (n = 13,714)

	**Increasing [a] mOR (95%CI)**	**Stable-medium [a] mOR (95%CI)**	**Stable-high [a] mOR (95%CI)**
**Age**	1.00 (0.95, 1.07)	1.01 (0.95, 1.07)	1.01 (0.93, 1.09)
**Education**			
Highschool or less	Reference	Reference	Reference
Tertiary not university	0.91 (0.72, 1.16)	0.89 (0.72, 1.10)	0.82 (0.57, 1.19)
College/ university	0.97 (0.73, 1.29)	0.95 (0.72, 1.24)	0.76 (0.48, 1.19)
**Living arrangement**			
Live alone	Reference	Reference	Reference
Live with others	1.04 (0.65, 1.66)	1.30 (0.82, 2.04)	0.98 (0.61, 1.58)
**Marital status**			
Partnered	Reference	Reference	Reference
Non-partnered	1.26 (0.96, 1.66)	1.23 (0.95, 1.60)	1.28 (0.92, 1.78)
Widowed	1.74 (1.00, 3.03)	1.53 (0.87, 2.66)	**2.08 (1.15, 3.78)**
**Currently employed**			
No	Reference	Reference	Reference
Yes	0.98 (0.78, 1.25)	0.96 (0.78, 1.18)	0.85 (0.64, 1.12)
**IRSD**	1.00 (1.00, 1.00)	1.00 (1.00, 1.00)	1.00 (1.00, 1.00)
**Location of residence**			
Major city	Reference	Reference	Reference
Regional	1.12 (0.86, 1.44)	1.03 (0.81, 1.31)	0.95 (0.71, 1.26)
Remote	0.95 (0.55, 1.64)	1.03 (0.69, 1.55)	1.37 (0.77, 2.42)
**Born in Australia**			
Yes	Reference	Reference	Reference
No	0.94 (0.69, 1.27)	1.01 (0.80, 1.28)	0.86 (0.62, 1.20)
**Language spoken at home**			

	Increasing [a] mOR (95%CI)	Stable-medium [a] mOR (95%CI)	Stable-high [a] mOR (95%CI)
English	Reference	Reference	Reference
European	1.18 (0.64, 2.17)	1.09 (0.69, 1.73)	1.14 (0.60, 2.15)
Asian	0.96 (0.30, 3.10)	0.89 (0.34, 2.32)	1.08 (0.30, 3.81)
Other	1.42 (0.54, 3.75)	1.00 (0.35, 2.83)	1.73 (0.51, 5.90)
**Risky alcohol consumption (>10 drinks p/week) [1]**			0.98 (0.64, 1.50)
No	Reference	Reference	Reference
Yes	1.26 (0.86, 1.87)	1.06 (0.78, 1.45)	0.98 (0.64, 1.50)
**Binge drinking (>1 episode p/week)**			
No	Reference	Reference	Reference
Yes	0.98 (0.74, 1.30)	1.12 (0.91, 1.39)	0.98 (0.72, 1.34)
**Smoking status**			
Non-smoker	Reference	Reference	Reference
Ex-smoker	1.03 (0.80, 1.34)	1.11 (0.91, 1.35)	1.05 (0.75, 1.45)
Current smoker	**1.28 (1.00, 1.62)**	**1.25 (1.00, 1.54)**	1.13 (0.80, 1.61)
**Body mass index**			
Healthy (18.5≤ BMI≤24.9	Reference	Reference	Reference
Underweight (<18.5)	0.83 (0.40, 1.73)	0.89 (0.41, 1.91)	1.41 (0.60, 3.32)
Overweight (25.0≤ BMI ≤29.9)	1.05 (0.83, 1.33)	1.12 (0.92, 1.37)	1.28 (0.94, 1.73)
Obese (≥30.0)	1.07 (0.82, 1.41)	1.19 (0.92, 1.52)	**1.55 (1.12, 2.15)**
**Depression (CES-D score)**	**1.07 (1.04, 1.11)**	**1.06 (1.03, 1.09)**	**1.11 (1.07, 1.15)**
**Stress score**	**1.33 (1.10, 1.62)**	**1.40 (1.13, 1.74)**	**1.72 (1.34, 2.20)**
**Depression [2]**			
No	Reference	Reference	Reference
Yes	**1.50 (1.11, 2.01)**	**1.31 (1.02, 1.70)**	1.34 (0.97, 1.85)
**Anxiety [3]**			
No	Reference	Reference	Reference
Yes	0.99 (0.77, 1.28)	1.06 (0.86, 1.31)	0.83 (0.60, 1.15)
**Social support score**	**0.70 (0.59, 0.83)**	**0.76 (0.64, 0.90)**	**0.55 (0.48, 0.62)**

Note.

Bold text indicates significant findings.

[a] Reference class: ‘stable low’. mORs refer to the odds of having the attribute in a certain class compared to the stable-low class.

Abbreviations: mOR: Multinomial odds ratio, CES-D: Center for Epidemiological Studies- Depression, IRSD: Index of Relative Socio-Economic Disadvantage

[1] Risky alcohol consumption: consuming more than 10 standard drinks in a week or more than four standard drinks per day.

[2] Depression: ever being told by a doctor about having depression or having symptoms of depression.

[3] Anxiety: ever being told by a doctor about having anxiety or having symptoms of anxiety.

Table S4.2. Sensitivity analysis: Multivariate multinomial linear regression predicting latent class membership using baseline predictors (Beta coefficients, 95%CI): Data fully adjusted, adjusted only for sociodemographic characteristics and unadjusted- Probit model results (n = 13,714)

		Increasing [a]	Stable-medium [a]	Stable-high [a]
		**B (95%CI)**	**B (95%CI)**	**B (95%CI)**
**PCS**	Fully adjusted	-0.80 (-1.92, 0.31)	**-1.09 (-2.17, -0.11)**	-0.78 (-1.87, 0.31)
	Sociodemographic adjusted	**-3.62 (-4.97, -2.26)**	**-3.73 (-5.10, -2.35)**	**-4.85 (-6.22, -3.49)**
	Unadjusted	**-4.10 (-5.51, -2.68)**	**-4.11 (-5.53, -2.68)**	**-5.58 (-6.99, -4.18)**
**MCS**	Fully adjusted	**-2.87 (-4.51, -1.24)**	**-3.97 (-5.40, -2.55)**	**-9.56 (-6.84, -4.49)**
	Sociodemographic adjusted	**-6.78 (-8.23, -5.33)**	**-7.17 (-8.23, -5.33)**	**-10.53 (-12.11, -8.95)**
	Unadjusted	**-7.05 (-8.54, -5.56)**	**-7.41 (-9.30, -5.50)**	**-10.93 (-12.53, -9.33)**
**Physical functioning**	Fully adjusted	-2.26 (-5.06, 0.55)	**-3.52 (-6.50, -0.54)**	**-4.32 (-7.04, -1.60)**
	Sociodemographic adjusted	**-9.08 (-12.45, -5.71)**	**-9.81 (-13.46, -6.15)**	**-14.00 (-17.18, -10.82)**
	Unadjusted	**-10.29 (-13.79, -6.80)**	**-10.79 (-14.55, -7.02)**	**-15.88 (-19.16, -12.60)**
**Role physical**	Fully adjusted	**-6.69 (-10.98, -2.41)**	**-8.57 (-13.57, -3.57)**	**-9.69 (-13.72, -5.66)**
	Sociodemographic adjusted	**-18.93 (-24.15, -13.72)**	**-19.38 (-25.64, -13.11)**	**-26.31 (-31.38, -21.24)**
	Unadjusted	**-20.53 (-26.00, -15.07)**	**-20.63 (-27.06, -14.20)**	**-28.65 (-33.85, -23.45)**
**Pain index**	Fully adjusted	**-3.23 (-6.05, -0.42)**	**-4.57 (-7.11, -2.03)**	**-4.67 (-6.96, -2.38)**
	Sociodemographic adjusted	**-10.76 (-13.70, -7.82)**	**-11.34 (-14.39, -8.29)**	**-14.97 (-18.04, -11.90)**
	Unadjusted	**-11.65 (-14.67, -8.64)**	**-12.06 (-15.19, -8.93)**	**-16.33 (-19.51, -13.14)**
**General health**	Fully adjusted	**-3.11 (-5.19, -1.03)**	**-4.18 (-7.00, -2.55)**	**-4.78 (-7.00, -2.55)**
	Sociodemographic adjusted	**-10.47 (-12.73, -8.21)**	**-10.59 (-13.38, -7.81)**	**-14.71 (-17.61, -11.81)**

	Unadjusted	**-11.31 (-13.73, -8.89)**	**-7.76 (-14.23, -8.33)**	**-15.98 (-18.95, -13.01)**
**Vitality**	Fully adjusted	**-3.65 (-7.01, -0.29)**	**-5.47 (-8.11, -2.83)**	**-7.20 (-9.28, -5.12)**
	Sociodemographic adjusted	**-12.42 (-15.50, -9.34)**	**-12.91 (-16.26, -9.56)**	**-18.67 (-21.48, -15.86)**
	Unadjusted	**-13.21 (-16.39, -10.03)**	**-13.56 (-17.04, -10.08)**	**-19.86 (-22.72, -17.00)**
**Social functioning**	Fully adjusted	**-6.11 (-9.47, -2.76)**	**-7.79 (-10.73, -4.86)**	**-10.68 (-13.47, -7.88)**
	Sociodemographic adjusted	**-14.39 (-17.93, -10.85)**	**-14.92 (-18.74, -11.10)**	**-21.58 (-24.98, -18.18)**
	Unadjusted	**-15.30 (-18.94, -11.65)**	**-15.64 (-19.59, -11.70)**	**-22.90 (-26.35, -19.45)**
**Role emotional**	Fully adjusted	**-7.60 (-12.66, -2.54)**	**-10.09 (-14.00, -6.18)**	**-15.24 (-19.86, -10.62)**
	Sociodemographic adjusted	**-17.09 (-22.21, -11.97)**	**-18.15 (-23.65, -12.65)**	**-27.47 (-32.97, -21.98)**
	Unadjusted	**-18.15 (-23.38, -12.92)**	**-19.04 (-24.79, -13.29)**	**-29.03 (-34.67, -23.39)**
**Mental health index**	Fully adjusted	**-4.82 (-7.51 (-2.13)**	**-6.97 (-9.62, -4.33)**	**-9.30 (11.35, -7.25)**
	Sociodemographic adjusted	**-11.80 (-14.21, -9.40)**	**-12.70 (-16.19, -9.22)**	**-18.00 (-20.78, -15.23)**
	Unadjusted	**-12.28 (-14.77, -9.80)**	**-13.12 (-16.71, -9.53)**	**-18.74 (-21.52, -15.95)**

Note.

Bold text indicates significant findings.

Reference group = 'Stable low' loneliness

Abbreviations: PCS: Physical Component Summary; MCS: Mental Component Summary; B: unstadardised Beta coefficients; M: Mean; SD: Standard deviation

Sociodemographic models were adjusted for age, gender, education, marital status, living arrangement, employment, area remoteness, country of birth and language spoken at home.

Fully adjusted models were adjusted for age, gender, education, marital status, living arrangement, employment, area remoteness, country of birth, language spoken at home, BMI, smoking, alcohol consumption, depression, anxiety, social support and stress.

Table S4.3. Sensitivity analysis: criteria for selecting the number of trajectories (waves 3-8) using a new cut-point for loneliness (1. Rarely or none of the time vs. levels 2-4; n = 13,714)

**Model**	**AIC**	**BIC**	**SSABIC**	**Modal group membership proportion**					
				(1)	(2)	(3)	(4)	(5)	(6)
**Overall loneliness**									
**1 class**	80636.62	80688.45	80666.20	100%					
**2 class**	69762.83	69873.89	69826.22	66.90%	33.10%				
**3 class**	68978.24	69148.53	69075.43	50.68%	35.91%	13.41%			
**4 class**	**68731.29**	**68960.82**	**68862.30**	**51.98%**	**13.07%**	**16.62%**	**18.32%**		
**5 class**	68708.33	68997.09	68873.15	37.53%	17.46%	15.95%	16.11%	12.94%	
**6 class**	68694.43	69035.01	68888.83	34.56%	15.13%	12.61%	11.43%	18.00%	8.27%

Bold text highlights the best model fit.

Abbreviations: AIC = Akaike information criterion; BIC = Bayesian information criterion; SSABIC = sample size adjusted BIC.

Table S4.4. Sensitivity analysis: Multivariate multinomial logistic regression predicting latent class membership using baseline predictors using a new cut-point for loneliness (1. Rarely or none of the time vs. levels 2-4; n = 13,714)

	**Increasing** [a] **mOR (95%CI)**	**Stable-medium** [a] **mOR (95%CI)**	**Stable-high** [a] **mOR (95%CI)**
Age	0.99 (0.93, 1.06)	0.98 (0.91, 1.06)	0.97 (0.91, 1.03)
Education			
Highschool or less	Reference	Reference	Reference
Tertiary not university	1.05 (0.88, 1.25)	0.95 (0.77, 1.18)	0.93 (0.76, 1.13)
College/ university	1.01 (0.81, 1.26)	0.95 (0.73, 1.24)	0.87 (0.68, 1.11)
Living arrangement			
Live alone	Reference	Reference	Reference
Live with others	1.01 (0.73, 1.39)	1.05 (0.73, 1.50)	0.96 (0.70, 1.32)
Marital status			
Partnered	Reference	Reference	Reference
Non-partnered	1.01 (0.64, 1.59)	1.28 (0.72, 2.29)	1.38 (0.95, 2.01)
Widowed	1.01 (0.40, 2.48)	1.10 (0.75, 1.62)	2.00 (0.94, 4.27)
Currently employed			
No	Reference	Reference	Reference
Yes	1.03 (0.86, 1.24)	1.02 (0.86, 1.21)	0.91 (0.76, 1.08)
IRSD	0.99 (0.99, 1.00)	1.00 (0.99, 1.00)	1.00 (1.00, 1.00)
Location of residence			
Major city	Reference	Reference	Reference
Regional	1.02 (0.80, 1.31)	1.10 (0.86, 1.41)	1.04 (0.83, 1.30)
Remote	1.01 (0.67, 1.51)	1.11 (0.75, 1.62)	1.33 (0.93, 1.92)
Born in Australia			
Yes	Reference	Reference	Reference
No	1.05 (0.86, 1.30)	1.08 (0.92, 1.26)	0.98 (0.80, 1.20)
Language spoken at home			
English	Reference	Reference	Reference
European	0.92 (0.62, 1.38)	0.95 (0.66, 1.37)	0.98 (0.67, 1.44)
Asian	0.68 (0.24, 1.94)	0.90 (0.48, 1.67)	1.16 (0.57, 2.33)

	**Increasing** [a] **mOR (95%CI)**	**Stable-medium** [a] **mOR (95%CI)**	**Stable-high** [a] **mOR (95%CI)**
Other	0.58 (0.17, 1.93)	1.09 (0.33, 3.61)	1.69 (0.82, 3.47)
Risky alcohol consumption (>10 drinks p/week) [1]			
No	Reference	Reference	Reference
Yes	1.06 (0.80, 1.40)	1.13 (0.86, 1.48)	1.01 (0.77, 1.31)
Binge drinking (>1 episode p/week)			
No	Reference	Reference	Reference
Yes	1.06 (0.91, 1.24)	0.98 (0.82, 1.17)	1.01 (0.84, 1.20)
Smoking status			
Non-smoker	Reference	Reference	Reference
Ex-smoker	1.03 (0.86, 1.25)	1.04 (0.89, 1.21)	1.08 (0.90, 1.31)
Current smoker	1.06 (0.80, 1.42)	1.11 (0.87, 1.40)	1.07 (0.84, 1.37)
Body mass index			
Healthy (18.5≤ BMI≤24.9	Reference	Reference	Reference
Underweight (<18.5)	0.64 (0.25, 1.63)	0.89 (0.49, 1.60)	0.90 (0.51, 1.60)
Overweight (25.0≤ BMI ≤29.9)	1.04 (0.90, 1.22)	0.89 (0.49, 1.60)	1.13 (0.95, 1.35)
Obese (≥30.0)	1.06 (0.87, 1.30)	1.03 (0.86, 1.23)	**1.31 (1.07, 1.59)**
Depression (CES-D score)	1.03 (0.92, 1.15)	1.05 (0.98, 1.13)	**1.10 (1.03, 1.17)**
Stress score	1.22 (0.62, 2.41)	1.34 (0.88, 2.05)	**1.63 (1.11, 2.38)**
Depression [2]			
No	Reference	Reference	Reference
Yes	1.00 (0.82, 1.24)	1.22 (0.84, 1.75)	**1.51 (1.08, 2.10)**
Anxiety [3]			
No	Reference	Reference	Reference
Yes	1.00 (0.82, 1.24)	1.08 (0.83, 1.40)	0.96 (0.78, 1.19)
Social support score	0.83 (0.46, 1.48)	0.78 (0.58, 1.05)	**0.58 (0.43, 0.79)**

Note.
Bold text indicates significant findings.

[a] Reference class: ‘Stable-low’. mORs refer to the odds of having the attribute in a certain class compared to the stable-low class.

Abbreviations: mOR: Multinomial odds ratio, CES-D: Center for Epidemiological Studies-Depression, IRSD: Index of Relative Socio-Economic Disadvantage

[1] Risky alcohol consumption: consuming more than 10 standard drinks in a week or more than four standard drinks per day.

[2] Depression: ever being told by a doctor about having depression or having symptoms of depression.

[3] Anxiety: ever being told by a doctor about having anxiety or having symptoms of anxiety.

Table S4.5. Sensitivity analysis: multivariate multinomial linear regression predicting latent class membership using baseline predictors (Beta coefficients, 95%CI) using a new cut-point for loneliness (1. Rarely or none of the time vs. levels 2-4): Data fully adjusted, adjusted only for sociodemographic characteristics and unadjusted (n = 13,714)

		**Increasing [a]**	**Stable-medium [a]**	**Stable-high [a]**
		**B (95%CI)**	**B (95%CI)**	**B (95%CI)**
**PCS**	Fully adjusted	-0.59 (-2.04, 0.86)	-0.63 (-1.32, 0.07)	**-1.07 (-2.08, -0.05)**
	Sociodemographic adjusted	-1.77 (-6.44, 2.89)	**-2.12, (-4.05, -0.18)**	**-4.56 (-6.94, -2.17)**
	Unadjusted	-1.87 (-6.90, 3.16)	**-2.29 (-4.44, -0.15)**	**-5.10, -7.68, -2.52)**
**MCS**	Fully adjusted	-2.56 (-6.85, 1.74)	**-1.53 (-2.51, -0.55)**	**-5.15 (-6.45, -3.83)**
	Sociodemographic adjusted	-4.00 (-12.65, 4.65)	**-3.46 (-5.05, -1.88)**	**-9.46 (-12.70, -6.22)**
	Unadjusted	-4.04 (-12.87, 4.78)	**-3.55 (-5.23, -1.86)**	**-9.70 (-13.03, -6.38)**
**Physical functioning**	Fully adjusted	-1.57 (-5.58, 2.45)	-1.67 (-3.49, 0.16)	**-4.31 (-6.95, -1.67)**
	Sociodemographic adjusted	-4.36 (-16.13, 7.4)	**-5.21 (-10.20, -0.22)**	**-12.59 (-18.51, -6.62)**
	Unadjusted	-4.58 (-17.25, 8.10)	**-5.64 (-11.18, -0.10)**	**-13.96 (-20.38, -7.55)**
**Role physical**	Fully adjusted	-5.68 (-16.35, 4.99)	**-4.46 (-7.04, -1.86)**	**-10.23 (-15.14, -5.32)**
	Sociodemographic adjusted	-10.45 (-34.96, 14.05)	**-10.69 (-17.93, -3.45)**	**-24.54 (-35.29, -13.78)**
	Unadjusted	-10.74 (-36.42, 14.93)	**-11.24 (-19.13, -3.36)**	**-26.20 (-37.61, -14.79)**
**Pain index**	Fully adjusted	-2.60 (-7.94, 2.73)	**-2.09 (-3.45, -0.74)**	**-4.92 (-7.34, -2.50)**
	Sociodemographic adjusted	-5.64 (-19.61, 8.34)	**-6.01 (-10.52, -1.50)**	**-13.87 (-20.13, -7.62)**
	Unadjusted	-5.81 (-20.44, 8.83)	**-6.31 (-11.16, 1.45)**	**-14.83 (-21.41, -8.24)**

**General health**	Fully adjusted	-2.56 (-7.23, 2.12)	**-1.66 (-2.99, -0.32)**	**-4.84 (-6.88, -2.81)**
	Sociodemographic adjusted	-5.48 (-18.55, 7.59)	-5.45 (-9.12, 1.78)	**-13.56 (-19.33, -7.79)**
	Unadjusted	-5.62 (-19.29, 8.05)	**-5.73 (-9.75, -1.71)**	**-14.47 (-20.56, -8.38)**
**Vitality**	Fully adjusted	-3.66 (-10.48, 3.15)	**-2.40 (-3.82, -0.99)**	**-6.54 (-9.09, -4.02)**
	Sociodemographic adjusted	-7.09 (-23.92, 9.74)	**-6.87 (-11.45, -2.29)**	**-16.72 (-23.80, 9.65)**
	Unadjusted	-7.24 (-24.65, 10.18)	**-7.14 (-12.04, -2.24)**	**-17.55 (-24.94, -10.16)**
**Social functioning**	Fully adjusted	-5.03 (-13.13, 3.06)	**-2.93 (-5.38, -0.48)**	**-9.88 (-13.02, -6.74)**
	Sociodemographic adjusted	-8.21 (-25.65, 9.24)	**-7.12 (-10.17, -4.06)**	**-19.37 (-26.55, -12.18)**
	Unadjusted	-8.37 (-26.53, 9.80)	**-7.44 (-10.88, -4.00)**	**-20.31 (-27.84, -12.77)**
**Role emotional**	Fully adjusted	-6.11 (-16.04, 3.82)	**-3.72 (-6.93, -0.52)**	**-13.29 (-17.25, -9.33)**
	Sociodemographic adjusted	-9.69 (-30.02, 10.65)	**-8.48 (-12.55, -4.40)**	**-24.00 (-31.82, -16.19)**
	Unadjusted	-9.88 (-30.94, 11.19)	**-8.84 (-13.28, -4.40)**	**-25.06 (-33.31, -16.80)**
**Mental health index**	Fully adjusted	-4.26 (-12.04, 3.52)	**-2.86 (-3.98, -1.74)**	**-9.00 (-11.74, -6.27)**
	Sociodemographic adjusted	-6.81 (-22.41, 8.78)	**-6.30 (-9.87, -2.73)**	**-16.65 (-23.00, -10.35)**
	Unadjusted	-6.89 (-22.77, 9.00)	**-6.43 (-10.15, -2.71)**	**-17.08 (-23.52, -10.64)**

* $p < 0.05$ ** $p < 0.01$ *** $p < 0.001$

Note.

Bold text indicates significant findings.

Reference group = ‘Stable-low’ loneliness

Abbreviations: PCS: Physical Component Summary; MCS: Mental Component Summary; B: unstadardised Beta coefficients; M: Mean; SD: Standard deviation

Sociodemographic models were adjusted for age, gender, education, marital status, living arrangement, employment, area remoteness, country of birth and language spoken at home.

Fully adjusted models were adjusted for age, gender, education, marital status, living arrangement, employment, area remoteness, country of birth, language spoken at home, BMI, smoking, alcohol consumption, depression, anxiety, social support and stress
